# Pretreatment SUV_max_ predicts progression-free survival in early-stage non-small cell lung cancer treated with stereotactic body radiation therapy

**DOI:** 10.1186/1748-717X-9-41

**Published:** 2014-01-30

**Authors:** Zachary D Horne, David A Clump, John A Vargo, Samir Shah, Sushil Beriwal, Steven A Burton, Annette E Quinn, Matthew J Schuchert, Rodney J Landreneau, Neil A Christie, James D Luketich, Dwight E Heron

**Affiliations:** 1 Department of Radiation Oncology, University of Pittsburgh Cancer Institute, 5230 Centre Ave, Pittsburgh PA 15232, USA; 2 Division of Thorcic and Foregut Surgery, Department of Cardiothoracic Surgery, University of Pittsburgh Medical Center, 200 Lothrop St, Suite C-816, Pittsburgh PA 15213, USA

## Abstract

**Background:**

This retrospective study aims to assess the usefulness of SUV_max_ from FDG-PET imaging as a prognosticator for primary biopsy-proven stage I NSCLC treated with SBRT.

**Methods:**

This study includes 95 patients of median age 77 years, with primary, biopsy-confirmed peripheral stage IA/IB NSCLC. All patients were treated with 60Gy in 3 fractions with a median treatment time of six days. Local, regional, and distant failures were evaluated independently according to the terms of RTOG1021. Local, regional, and distant control, overall- and progression-free survival were estimated by the Kaplan-Meier method. Cox proportional hazards regression was performed to determine whether SUV_max_, age, KPS, gender, tumor size/T stage, or smoking history influenced outcomes. SUV_max_ was evaluated as both a continuous and as a dichotomous variable using a cutoff of <5 and ≥5.

**Results:**

Median follow-up for the cohort was 16 months. Median OS and PFS were 25.3 and 40.3 months, respectively. SUV with a cutoff value of 5 predicted for OS and PFS (p = .024 for each) but did not achieve significance for LC (p = .256). On Cox univariate regression analysis, SUV as a dichotomous variable predicted for both OS and PFS (p = .027 and p = .030, respectively). Defined as a continuous variable, SUV_max_ continued to predict for OS and PFS (p = .032 and p = .003), but also predicted LC (p = .045) and trended toward significance for DC (p = .059).

SUV_max_ did not predict for OS as a dichotomous or continuous variable. It did, however, predict for PFS as a continuous variable (p = .008), neared significance for local control (p = .057) and trended towards, significance for distant control (p = .092).

**Conclusions:**

SUV_max_ appears to be a statistically and clinically significant independent prognostic marker for progression-free survival in patients with stage I NSCLC treated with SBRT. Prospective studies to more accurately define the role of tumor FDG uptake in the prognosis of NSCLC are warranted.

## Introduction

[^18^ F]-Fluorodeoxyglucose positron emission tomography (FDG-PET) is an important tool in the initial staging and subsequent assessment of patients diagnosed and treated for non-small cell lung cancer (NSCLC) [1,2]. FDG-PET imaging relies on the functional properties that define malignancies including increased glucose metabolism. This uptake is linked to tumor proliferation and metastatic potential and recent investigations demonstrate the usefulness of PET imaging as a prognosticator for eventual outcomes.

The International Association for the Study of Lung Cancer (IASLC) reviewed 21 studies that assessed the utility of the maximum standardized uptake value (SUV_max_) in NSCLC and determined that tumors with higher SUV_max_ have poorer prognoses [3]. Other recent studies have attempted to determine the utility of SUV_max_ under a more narrow scope including that of early-stage NSCLC treated with stereotactic body radiation therapy (SBRT), an emerging technique typically reserved for patients who are medically-inoperable or who refuse surgery [4,5]. Multiple studies demonstrate that pretreatment SUV_max_ predicts for clinical outcomes in patients with early-stage NSCLC treated with SBRT [6-8]. To the contrary, studies from Cleveland Clinic and Indiana University failed to find a correlation between pre-treatment SUV_max_ and survival [9,10]. As early-stage NSCLC is a potentially curable disease with SBRT, here, an SUV_max_ cutoff that predicts for more aggressive disease in patients with solitary, peripheral, primary stage I NSCLC is identified.

## Methods and materials

### Patients and workup

This study includes 95 non-consecutive patients treated for biopsy-confirmed peripheral stage IA/IB between October 2005 and May 2011 [11]. This research was determined to have exemption status by our Institutional Review Board. All patients were staged according to the 7^th^ edition of the AJCC criteria. No tumor was located within 2 cm of the proximal bronchial tree and no patient was previously treated for lung cancer. All patients had a pre-SBRT FDG-PET-CT scan with a documented SUV_max_. Of these patients, 14 were operable candidates but refused surgical therapy, while the remaining 81 patients had significant pulmonary or cardiac comorbidity that precluded definitive surgical management (Table 1). As a part of the staging, all patients underwent a PET-CT scan. The SUV_max_ was obtained from review of the formally dictated radiology report.

**Table 1:** Patient characteristics

**n = 95**	**Median**
Age	77 (48-91) years
Sex	
Male	49 (51.6%)
Female	46 (48.4%)
Operable	14 (14.7%)
Inoperable	81 (85.3%)
KPS	
80-100	63 (66.3%)
<70	32 (32.7%)
Clinical follow-up	16.33 (1.13-64.2) months

### Simulation and treatment

Each patient was positioned supine with arms raised above the head for the CT simulation. A thin-slice 4-D high resolution CT (2.5 mm) and 1.25 mm helical CT with intravenous contrast was obtained while the patient was immobilized in a custom BodyFIX vacuum bag (Electa). For patients treated with CyberKnife™, Synchrony Respiratory Tracking System (Accuray, Inc, Sunnyvale, CA) was utilized in conjunction with the 4D-CT to ensure fiducial movement in sync with the GTV. For Trilogy™ and Trubeam™ patients, image-guided respiratory cycle motion was accounted for via Varian Real-Time Position Management System (Varian Medical Systems, Palo Alto, CA). Respiratory gating was incorporated for patients with tumor motion > 0.5 cm. The acquired images were then transferred to the treatment planning workstation using either Accuray MulitPLAN™ (Accuray, Inc, Sunnyvale, CA) or Varian Eclipse™ (Varian Medical Systems, Palo Alto, CA). The AAA planning algorithm was utilized for patients treated on Trilogy™ and Trubeam™ and the pencil beam algorithm for patients treated on CyberKnife™. The tumor volume and any surrounding critical structures, including the spinal cord, heart, esophagus, brachial plexus and normal lung, were manually delineated by a radiosurgical team consisting of a radiation oncologist, a medical physicist, and a thoracic surgeon. The gross tumor volume (GTV) was defined as the tumor alone. To account for setup error and residual motion detected on end-exhalation 4D-CT, a minimum expansion of 5 mm margin was added to create the planning target volume (PTV). An additional margin based on motion assessment was added to create an internal target volume (ITV) to be used with gating. Dose-volume histograms were calculated for the target volume and nearby critical structures to select the optimal treatment plan, which provided at least 95% of the prescription dose to the PTV while sparing surrounding organs-at-risk. If surrounding organs-at-risk were deemed to be at excess risk for toxicity, a plan with lower PTV coverage was accepted.

SBRT was performed using CyberKnife™ Robotic Radiosurgery System (Accuray, Inc, Sunnyvale, CA for 39 patients, Trilogy™ Radiosurgery System (Varian Medical Systems, Palo Alto, CA) for 54 patients and Trubeam™ Radiosurgery System (Varian Medical Systems, Palo Alto, CA) for 2 patients. All lesions were treated with heterogeneity correction to 60 Gy in 3 fractions every other day with a median of 6 elapsed days from beginning of treatment to end (range 3-21 days). For patients treated on the Trilogy™ and Trubeam ™ platforms, cone-beam CT (CBCT) was performed daily to separate setup error from tumor reposition error. The treating physician checked and modified the alignment based on target relocalization in the fused imaging.

### Disease assessment and clinical follow-Up

After treatment, patients were scheduled to have either a CT or PET/CT scan every 3 months with a clinical evaluation. Response to treatment was evaluated by the RECIST v1.1 criteria and documented as a complete response, partial response (greater than 30% decrease in the longest axis), progressive disease (greater than 20% increase in the longest axis), or stable disease (neither partial response nor progressive disease) [12]. Follow-up imaging was re-evaluated to classify local, regional, and distant failures similar to the definitions of RTOG 1021 [13]. Local failures were defined as recurrence within the originally involved lobe or within 2 cm of the initial primary but located outside the originally involved lobe. Regional failure included non-involved ipsilateral lobes, as well as ipsilateral hilar, mediastinal, and subcarinal lymph nodes. Distant failures enveloped ipsilateral supraclavicular and contralateral lymph nodes and all other distant sites. Progression-free survival was defined as the time to a specified recurrence and was measured from the last day of treatment to that event. Death was not included as an endpoint for PFS. Local, regional, and distant control, overall- and progression-free survival were estimated by the Kaplan-Meier method. The ANOVA test was utilized to determine correlations between SUV_max_, tumor histology, and stage. Forward conditional Cox proportional hazards regression was performed to determine whether SUV_max_ (continuous/dichotomous), age (continuous), KPS (continuous), gender, tumor T stage, tumor histology, or smoking pack years (continuous) influenced outcomes. SUV_max_ was evaluated in univariate and multivariate analyses as both a continuous and as a dichotomous variable using a cutoff of <5 and ≥5 as described in previous reports [6,9,14,15]. All statistics were completed using SPSS version 20 (IBM Corp, Armonk, NY). Significance was set at p ≤ 0.05.

## Results

A total of 95 patients with a median age 77 years (range: 48-91 years) were identified between October 2005 and May 2011 (Table 1). All patients had biopsy-confirmed NSCLC, with 38 (40%) having squamous cell carcinoma and 33 (34.7%) having adenocarcinoma. The remaining 24 (25.3%) were not differentiated beyond NSCLC NOS. The median tumor size was 2.15 cm (range: 0.8-5.0 cm). Tumor T-stage distribution was, according to the AJCC 7^th^ edition, as follows: T1a: 46 (48.4%), T1b: 30 (31.6%), and T2a: 19 (20%) (Table 2). The median pretreatment SUV_max_ was 6.6 (range: 1.2-26.1). Among the 95 patients, 90 had follow-up imaging available (median number of scans: 2) for review with a median follow-up time of 16 months (range: 1-63 months). Imaging was performed to assess changes in tumor size, to identify development of additional tumors, and to evaluate effects on normal tissues. Of the patients who had follow-up imaging, 82 had at least one PET-CT scan.

**Table 2:** **Imaging and tumor characteristics with percentage distribution per SUV**_**max **_**category**

	**N (%)**	**Pretreatment PET SUV**_**max**_		
		**SUV**_**max**_ **< 5**	**SUV**_**max**_ **≥ 5**	***p***
All patients	95	40	55	ns
Histology				
Squamous	38 (40%)	8 (20%)	30 (54.5%)	**.046**
Adenocarcinoma	33 (34.7%)	21 (52.5%)	12 (21.8%)	
NSCLC NOS	24 (25.3%)	11 (27.5%)	13 (23.7%)	
Tumor Size [median (range)]	2.15 (0.8-5.0) cm	1.95 (0.9-5.0) cm	2.4 (0.8-4.8) cm	**.022**
T Stage				
1a	46 (48.4%)	27 (67.5%)	19 (34.5%)	**.013**
1b	30 (31.6%)	7 (17.5%)	23 (41.8%)	
2a	19 (20%)	6 (15%)	13 (23.7%)	

Tumor demographics and SUV_max_ distributions showing tendency for squamous cell histology and larger size to be of increasing SUV_max_.

### PET response

Of the 82 who had at least one PET scan in follow-up, at first PET-CT scan, 6 achieved complete responses, 56 a partial response, 24 stable disease, and 2 progressive disease. During interval follow-up, the best response observed was: 21 complete responders, 49 partial responders, 19 with stable disease, and none with progressive disease.

### Toxicity

In the 95 patients treated with SBRT, three acute Grade 3 toxicities were observed. The Grade 3 toxicities were comprised of radiation pneumonitis, pneumonia, and pleural effusion. Additionally, there was one Grade 2 dyspnea and two Grade 2 chest pains. Late toxicities included one Grade 3 dyspnea and one Grade 2 dyspnea.

### Clinical outcomes

**Figure 1:**
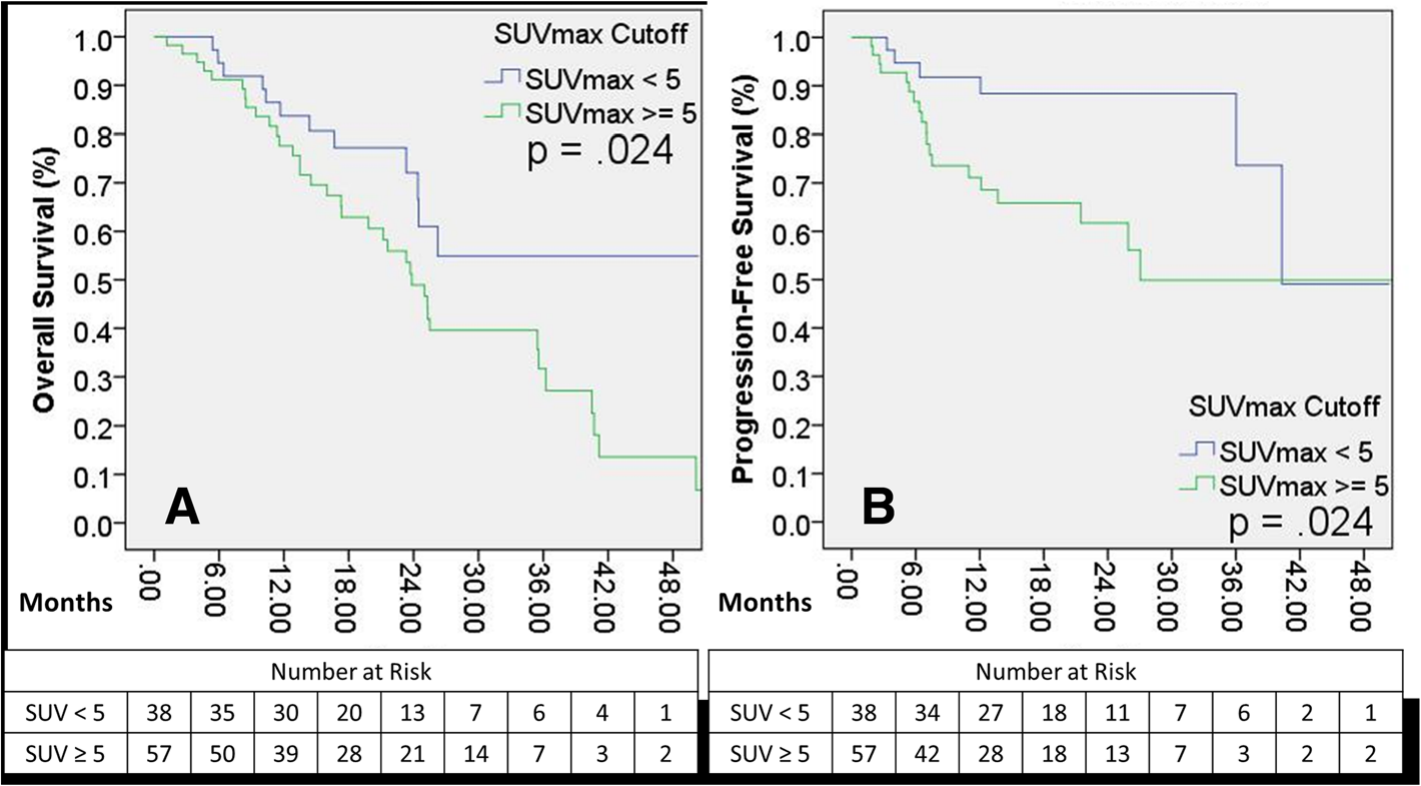
**Overall and progression-free survivals as differentiated by SUV**_**max**_**. A**: Overall survival differences between SUV_max_ categories, p= 0.024; **B**: Progression-free survival differences between SUV_max_ categories, p= 0.024.

**Figure 2:**
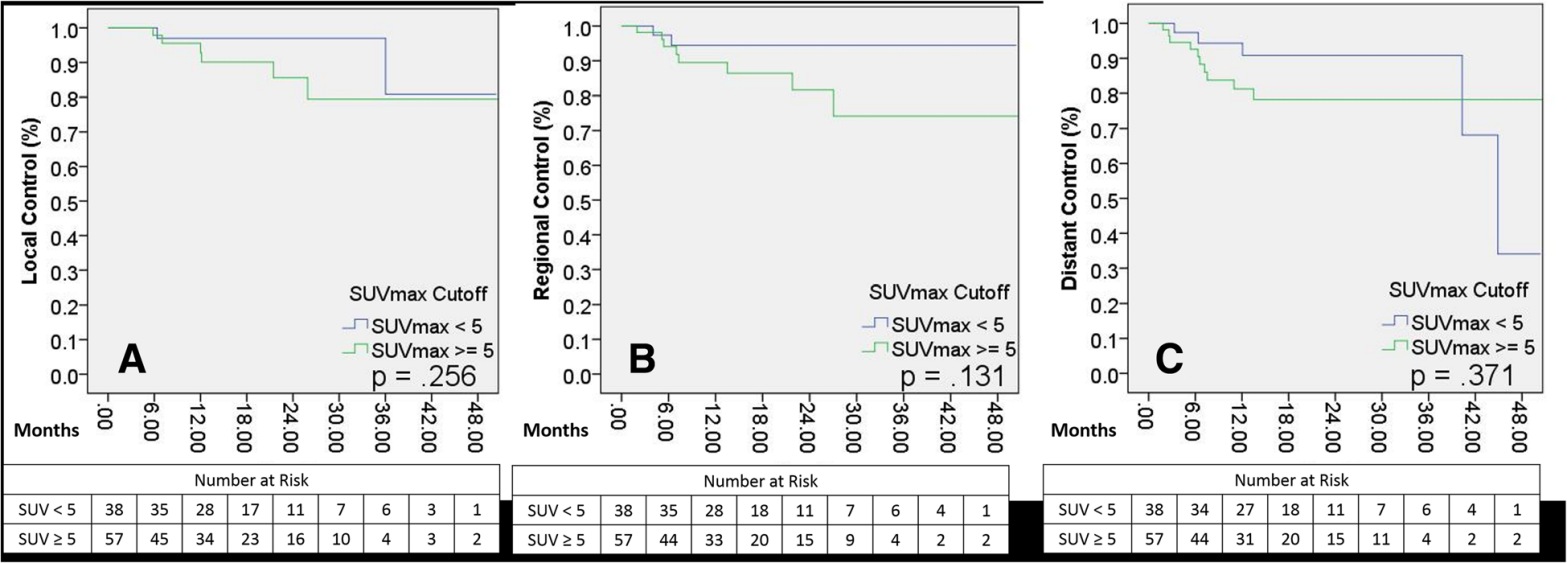
**Local, regional, and distant control rates as differentiated by SUV**_**max**_**. A**: Local control differences between SUV_max_ categories, p= 0.256; **B**: Regional control differences between SUV_max_ categories, p= 0.131; **C**: Distant control differences between SUV_max_ categories, p= 0.371.

Median overall survival (OS) and progression-free survival (PFS) were 25.3 and 40.3 months, respectively. The 2-year actuarial rates of events following treatment are shown in Table 3. Two-year overall and cause-specific survivals were 64.2% and 94.5%, respectively. Overall PFS at two years was 93.7%. Two-year local control was 93.7%, regional control was 90.5%, and distant control was 86.3%. For dichotomous variable analyses, an SUV of 5 was utilized as a cutoff as stated above. In univariate Kaplan-Meier analysis, OS was predicted by TNM T-stage (p = .007). There was no difference in survival between operable and non-operable patients (p = .313) or tumor histology (p = .292). SUV_max_ predicted for OS and PFS (p = .024 for each, Figures 1A, B) but did not achieve significance for local control (LC) (p = 0.256), regional control (RC) (p = 0.131), or distant control (DC) (p = 0.371) (Figures 2A, B, C). On Cox univariate regression analysis, SUV_max_ as a dichotomous variable predicted for both OS (p = .027, HR = 0.478) and PFS (p = .030, HR = 0.359). OS was also predicted by Karnofsky Performance Status (p < .0001) and TNM T-stage (p = .005). Defined as a continuous variable, SUV_max_ continued to predict for OS (p = .032, HR = 1.061) and PFS (p = .003, HR = 1.098), but also achieved significance for LC (p = .045, HR = 1.124) and trended toward significance for DC (p = .059) (Table 4). On ANOVA test, tumor T-stage and histology were both significantly correlated to SUV_max_ (p = .046 and p = .013, respectively).

**Table 3:** **Overall outcomes from treatment with 2-year event rates showing differences between SUV**_**max **_**categories**

			**SUV**_**max**_** < 5**	**SUV**_**max**_ **≥ 5**	
	**2-year freedom from event (%)**	**Total events n (%)**	**2-year freedom (%)**	**2-year freedom (%)**	**K-M p**
Local failure	93.7	8 (8.4)	97	86	.256
Regional failure	90.5	10 (10.5)	94	82	.131
Distant failure	86.3	15 (15.8)	91	78	.371
Any progression	93.7	25 (26.3)	88	62	**.024**
Death	64.2	48 (50.5)	72	49	**.024**

Comparison of SUV_max_ categories shows a statistically significant difference in progression-free and overall survivals.

**Table 4:** **Univariate and multivariate Cox proportional hazards regression analysis with SUV**_**max **_**as a dichotomous and continuous variable**

**Univariate Cox proportional hazards regression analysis**				
	**PET SUV**_**max **_**(<5 vs ≥5)**		**PET SUV**_**max **_**(Continuous)**	
	**HR (95% CI)**	**p**	**HR (95% CI)**	**p**
Local control	--	NS	1.124 (1.002 – 1.260)	**.045**
Distant control	--	NS	--	.059
Progression-free survival	0.359 (0.143 – 0.905)	**.030**	1.098 (1.033 – 1.168)	**.003**
Overall survival	0.478 (0.249 – 0.920)	**.027**	1.061 (1.005 – 1.119)	**.032**
Multivariate Cox proportional hazards regression analysis				
Local control	--	NS	--	.057
Distant control	--	NS	--	.092
Progression-free survival	--	.105	1.111 (1.027 – 1.201)	**.008**

Univariate Cox hazards regression analysis of SUV_max_ as a dichotomous variable shows significance for progression-free and overall survivals. As a continuous variable, SUV_max_ significantly predicts for local control, progression-free and overall survivals. In multivariate Cox analysis, SUV_max_ remains a significant predictor of progression-free survival.

In multivariate analysis, SUV_max_ did not predict for OS as a dichotomous or continuous variable and only KPS and T-stage remained significant (p < .0001 and p = .013, respectively). It did, however, predict for PFS as a continuous variable (p = .008, HR = 1.111), though not as a dichotomous variable (Table 4). SUV_max_ also trended toward significance for LC and DC as a continuous variable (p = .057 and p = .092, respectively).

## Discussion

Previous reports indicate that tumor aggressiveness defined by an increased Ki-67 is correlated with SUV_max_ and tumor differentiation but not with TNM stage [16,17]. Lung tumors with high Ki-67 are associated with decreased survival [18] as well as shortened progression-free survival in surgical series [19,20]. This would indicate that tumors with higher SUV_max_ values have an increased likelihood of having a more aggressive biology regardless of size which in our study, manifests in decreased progression-free survival.

Our study of patients with primary, peripheral, biopsy-proven, stage I NSCLC treated with a homogenous SBRT regimen found that SUV_max_ predicts for progression-free survival. OS was predicted by tumor stage, a finding that is expected. When analyzing SUV cutoffs for significance, a range of SUV values showed significant results for different endpoints. A meta-analysis of surgical studies utilizing differing SUV cutoffs by Paesmans et al also found that SUV_max_ is a significant prognosticator for overall survival [3].

To date, similar studies on patients treated with either conventionally fractionated radiation therapy or SBRT have been unable to come to consensus on the utility of SUV_max_ as a prognosticator [6-10,15,21]. Ikushima et al’s study of definitive external beam radiation found SUV_max_ to be related to tumor size/stage but not outcomes and hypothesized that the result was due to partial volume effect (PVE) as outlined by Soret et al [21,22]. In this study, we also found SUV_max_ to be related to tumor size (p = .013), but tumor size did not predict for the same outcomes as SUV_max_. The results from Ikushima et al’s study are in contrast to the results from the Sasaki group, which showed an SUV cutoff of 5 to be significant for OS and PFS in patients treated with conventional RT [15]. In the two studies which investigated SUV_max_ as a prognostic factor for the treatment of early-stage NSCLC with SBRT and failed to achieve significance, both cite sample size as a possible contributor to the lack of findings [9,10]. The studies that did find significance in SUV_max_ as a predictor of outcomes also suffer from problems such as heterogeneous treatment dosing, tumor staging and location, and sample size [6-8]. With a reduction in confounding variables by evaluating patients with a uniformity in stage, treatment dose and fractionation, and confirmation that all patients do indeed have non-small cell lung cancer, this study is able to provide a more focused insight into the prognostic value of SUV_max_. An additional study by Abelson et al. infers that the amount of metabolically active tumor may be equally important to outcomes as the peak metabolic activity of the tumor and warrants further investigation to elucidate the relationship between the two [23].

To better determine the external validity of the results of this study, the most appropriate SUV_max_ cutoff needs to be generated from pooled data from multiple high-volume centers. To determine the applicability of this information to the general stage 1 SBRT lung population, this data needs to include patients without pathological confirmation of disease as well as those who were treated under alternative fractionation schedules. Additionally, studies that include medically operable patients will provide longer-term data than current studies which rely on inoperable patients, most of whom succumb to intercurrent disease. At the time of analysis in this study, the cause of death in at least 56% of the patients who had perished was attributable to intercurrent disease. Armed with the upfront knowledge that some patients have more aggressive disease than others, as well as the emerging evidence that early post-treatment FDG-PET scanning may allow further differentiation of at-risk patients [6], clinical trials may emerge to offer treatment-intensification.

There are several limitations to this study including size, limited follow-up, its retrospective nature, and the inherent variability of FDG-PET scans and SUV_max_ measurements from machine to machine and interobserver variability. A multi-institutional prospective study which utilizes a standardized protocol for administering and reading FDG-PET scans as well as biopsy information to correlate tumor biology to scan information and outcomes is likely necessary to confirm the findings found herein.

SUV_max_ appears to be a useful prognosticator for progression-free survival and overall survival in the therapy of early-stage NSCLC treated with SBRT. As a predictor in both dichotomous and continuous forms, SUV_max_ seems to be correlated with the propensity of tumors to metastasize. Larger studies may reveal a more appropriate cutoff value for identifying patients with more aggressive disease, which may then provide the basis for clinical trials to identify the benefit of more vigorous therapy.

## Competing interest

The authors’ declare that they have no competing interest.

## Authors’ contributions

ZDH, DAC, JAV, DEH drafted the manuscript. SS, SB, SAB, AEQ, MJS, RJL, NAC, JDL gathered data and edited the manuscript. All authors read and approved the final manuscript.

## References

[B1] Kligerman S Digumarthy S Staging of non-small cell lung cancer using integrated PET/CT AJR Am J Roentgenol 2009 193 5 1203 1211 10.2214/AJR.09.3193 19843732

[B2] Mac Manus MP Hicks RJ The role of positron emission tomography/computed tomography in radiation therapy planning for patients with lung cancer Semin Nucl Med 2012 42 5 308 319 10.1053/j.semnuclmed.2012.04.003 22840596

[B3] Paesmans M Berghmans T Dusart M Primary tumor standardized uptake value measured on fluorodeoxyglucose positron emission tomography is of prognostic value for survival in non-small cell lung cancer: update of a systematic review and meta-analysis by the European Lung Cancer Working Party for the International Association for the Study of Lung Cancer Staging Project J Thorac Oncol: Offic Publ Int Assoc Stud Lung Canc 2010 5 5 612 619 10.1097/JTO.0b013e3181d0a4f5 20234323

[B4] Onishi H Shirato H Nagata Y Hypofractionated stereotactic radiotherapy (HypoFXSRT) for stage I non-small cell lung cancer: updated results of 257 patients in a Japanese multi-institutional study J Thorac Oncol: Offic Publ Int Assoc Stud Lung Canc 2007 2 7 Suppl 3 S94 S100 10.1097/JTO.0b013e318074de34 17603311

[B5] Timmerman R Paulus R Galvin J Stereotactic body radiation therapy for inoperable early stage lung cancer JAMA: J Am Med Assoc 2010 303 11 1070 1076 10.1001/jama.2010.261 PMC2907644 20233825

[B6] Clarke K Taremi M Dahele M Stereotactic body radiotherapy (SBRT) for non-small cell lung cancer (NSCLC): is FDG-PET a predictor of outcome? Radiother Oncol: J Eur Soc Ther Radiol Oncol 2012 104 1 62 66 10.1016/j.radonc.2012.04.019 22682749

[B7] Hamamoto Y Sugawara Y Inoue T Relationship between pretreatment FDG uptake and local control after stereotactic body radiotherapy in stage I non-small-cell lung cancer: the preliminary results Jpn J Clin Oncol 2011 41 4 543 547 10.1093/jjco/hyq249 21262874

[B8] Takeda A Yokosuka N Ohashi T The maximum standardized uptake value (SUVmax) on FDG-PET is a strong predictor of local recurrence for localized non-small-cell lung cancer after stereotactic body radiotherapy (SBRT) Radiother Oncol: J Eur Soc Ther Radiol Oncol 2011 101 2 291 297 10.1016/j.radonc.2011.08.008 21889224

[B9] Burdick MJ Stephans KL Reddy CA Djemil T Srinivas SM Videtic GM Maximum standardized uptake value from staging FDG-PET/CT does not predict treatment outcome for early-stage non-small-cell lung cancer treated with stereotactic body radiotherapy Int J Radiat Oncol Biol Phys 2010 78 4 1033 1039 10.1016/j.ijrobp.2009.09.081 20472359

[B10] Hoopes DJ Tann M Fletcher JW FDG-PET and stereotactic body radiotherapy (SBRT) for stage I non-small-cell lung cancer Lung Canc 2007 56 2 229 234 10.1016/j.lungcan.2006.12.009 17353064

[B11] Edge SB, Byrd DR, Compton CC, Fritz AG, Greene FL, Trotti A AJCC cancer staging manual (7th ed) 2010 New York, NY: Springer

[B12] Eisenhauer EA Therasse P Bogaerts J New response evaluation criteria in solid tumours: revised RECIST guideline (version 1.1) Eur J Canc 2009 45 2 228 247 10.1016/j.ejca.2008.10.026 19097774

[B13] Radiation Therapy Oncology Group Radiation Therapy Oncology Group 1021 Protocol 2012

[B14] Detterbeck FC Vansteenkiste JF Morris DE Dooms CA Khandani AH Socinski MA Seeking a home for a PET, part 3: emerging applications of positron emission tomography imaging in the management of patients with lung cancer Chest 2004 126 5 1656 1666 10.1378/chest.126.5.1656 15539740

[B15] Sasaki R Komaki R Macapinlac H [18F]fluorodeoxyglucose uptake by positron emission tomography predicts outcome of non-small-cell lung cancer J Clin Oncol: Offic J Am Soc Clin Oncol 2005 23 6 1136 1143 10.1200/JCO.2005.06.129 15718309

[B16] Vesselle H Salskov A Turcotte E Relationship between non-small cell lung cancer FDG uptake at PET, tumor histology, and Ki-67 proliferation index J Thorac Oncol: Offic Publ Int Assoc Stud Lung Canc 2008 3 9 971 978 10.1097/JTO.0b013e31818307a7 18758298

[B17] Vesselle H Schmidt RA Pugsley JM Lung cancer proliferation correlates with [F-18]fluorodeoxyglucose uptake by positron emission tomography Clin Canc Res: Offic J Am Assoc Canc Res 2000 6 10 3837 3844 11051227

[B18] Martin B Paesmans M Mascaux C Ki-67 expression and patients survival in lung cancer: systematic review of the literature with meta-analysis Brit J Canc 2004 91 12 2018 2025 10.1038/sj.bjc.6602233 PMC2409786 15545971

[B19] Woo T Okudela K Yazawa T Prognostic value of KRAS mutations and Ki-67 expression in stage I lung adenocarcinomas Lung Canc 2009 65 3 355 362 10.1016/j.lungcan.2008.11.020 19162366

[B20] Mehdi SA Tatum AH Newman NB Prognostic markers in resected stage I and II non small-cell lung cancer: an analysis of 260 patients with 5 year follow-up Clin Lung Canc 1999 1 1 59 67 discussion 68-59 10.3816/CLC.1999.n.004 14725752

[B21] Ikushima H Dong L Erasmus J Predictive value of 18 F-fluorodeoxyglucose uptake by positron emission tomography for non-small cell lung cancer patients treated with radical radiotherapy J Radiat Res 2010 51 4 465 471 10.1269/jrr.10024 20508373

[B22] Soret M Bacharach SL Buvat I Partial-volume effect in PET tumor imaging J Nucl Med: Offic Publ Soc Nucl Med 2007 48 6 932 945 10.2967/jnumed.106.035774 17504879

[B23] Abelson JA Murphy JD Trakul N Metabolic imaging metrics correlate with survival in early stage lung cancer treated with stereotactic ablative radiotherapy Lung Canc 2012 78 3 219 224 10.1016/j.lungcan.2012.08.016 23009727

